# *N*-(4-Isopropoxyphen­yl)acetamide

**DOI:** 10.1107/S1600536809012665

**Published:** 2009-04-10

**Authors:** Min Zhang, Ran-Zhe Lu, Lu-Na Han, Wen-Bin Wei, Hai-Bo Wang

**Affiliations:** a College of Light Industry and Food Science, Nanjing University of Technology, Xinmofan Road No.5 Nanjing, Nanjing 210009, People’s Republic of China; b College of Science, Nanjing University of Technology, Xinmofan Road No.5 Nanjing, Nanjing 210009, People’s Republic of China

## Abstract

In the mol­ecule of the title compound, C_11_H_15_NO_2_, the planar acetamide unit [maximum deviation of 0.0014 (6) Å] is oriented at a dihedral angle of 19.68 (4)° with respect to the aromatic ring. An intra­molecular C—H⋯O inter­action results in the formation of a six-membered ring. In the crystal structure, inter­molecular N—H⋯O hydrogen bonds link the mol­ecules into chains along the *a* axis

## Related literature

For general background, see: Knesl *et al.* (2006 ▶). For bond-length data, see: Allen *et al.* (1987 ▶).
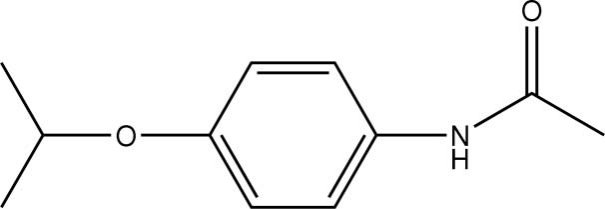


## Experimental

### 

#### Crystal data

C_11_H_15_NO_2_
*M* *_r_* = 193.24
Orthorhombic, 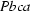

*a* = 9.3010 (19) Å
*b* = 7.6490 (15) Å
*c* = 31.394 (6) Å
*V* = 2233.5 (8) Å^3^
*Z* = 8
Mo *K*α radiation
μ = 0.08 mm^−1^
*T* = 294 K
0.30 × 0.10 × 0.10 mm

#### Data collection

Enraf–Nonius CAD-4 diffractometer
Absorption correction: ψ scan (North *et al.*, 1968 ▶) *T*_min_ = 0.977, *T*_max_ = 0.992
2026 measured reflections
2026 independent reflections
1099 reflections with *I* > 2σ(*I*)
3 standard reflections frequency: 120 min intensity decay: 1%

#### Refinement

*R*[*F* ^2^ > 2σ(*F* ^2^)] = 0.068
*wR*(*F* ^2^) = 0.202
*S* = 1.01
2026 reflections
127 parameters
H-atom parameters constrained
Δρ_max_ = 0.26 e Å^−3^
Δρ_min_ = −0.23 e Å^−3^

### 

Data collection: *CAD-4 Software* (Enraf–Nonius, 1989 ▶); cell refinement: *CAD-4 Software*; data reduction: *XCAD4* (Harms & Wocadlo, 1995 ▶); program(s) used to solve structure: *SHELXS97* (Sheldrick, 2008 ▶); program(s) used to refine structure: *SHELXL97* (Sheldrick, 2008 ▶); molecular graphics: *PLATON* (Spek, 2009 ▶) and *ORTEP-3* (Farrugia, 1997 ▶); software used to prepare material for publication: *SHELXTL* (Sheldrick, 2008 ▶).

## Supplementary Material

Crystal structure: contains datablocks D, I. DOI: 10.1107/S1600536809012665/hk2659sup1.cif

Structure factors: contains datablocks I. DOI: 10.1107/S1600536809012665/hk2659Isup2.hkl

Additional supplementary materials:  crystallographic information; 3D view; checkCIF report

## Figures and Tables

**Table 1:** Hydrogen-bond geometry (Å, °)

*D*—H⋯*A*	*D*—H	H⋯*A*	*D*⋯*A*	*D*—H⋯*A*
N—H0*A*⋯O2^i^	0.86	2.01	2.869 (3)	175
C6—H6*A*⋯O2	0.93	2.34	2.892 (4)	118

Symmetry code: (i) 
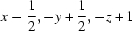
.

**Fig. 1.**
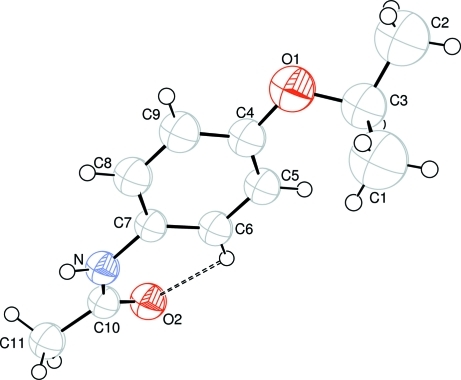
The molecular structure of the title molecule, with the atom-numbering scheme. Hydrogen bond is shown as dashed line.

## supplementary crystallographic information

### Comment

As part of our ongoing studies on tandutinib (Knesl *et al.*, 2006), we report herein the crystal structure of the title compound.

In the molecule of the title compound (Fig 1), the bond lengths (Allen *et al.*,  1987) and angles are within normal ranges. Ring A (C4-C9) is, of course, planar. The B (O2/N/C10/C11) moiety is also planar with a maximum deviation of -0.0014 (6) Å for C10 atom, and it is oriented with respect to ring A at a dihedral angle of 19.68 (4)°. Intramolecular C-H···O interaction (Table 1) results in the formation of a six-membered ring C (O2/N/C6/C7/C10/H6A), having twisted conformation.

In the crystal structure, intermolecular N-H···O hydrogen bonds (Table 1) link the molecules into chains along the a axis, in which they may be effective in the stabilization of the structure.

### Experimental

For the preparation of the title compound, N-(4-hydroxyphenyl)acetamide (50 mmol), 2-bromopropane (75 mmol) and potassium hydroxide (100 mmol) were mixed with ethanol (60 ml), and then the mixture was heated to reflux. Reaction progress was monitored by TLC. After ethanol removed in vacuo and filtration, the title compound was obtained (yield; 83.2%, m.p. 403 K). Crystals suitable for X-ray analysis were obtained by slow evaporation of an ethyl acetate solution.

### Refinement

H atoms were positioned geometrically, with N-H = 0.86 Å (for NH) and C-H = 0.93, 0.98 and 0.96 Å for aromatic, methine and methyl H, respectively, and constrained to ride on their parent atoms, with U_iso_(H) = xU_eq_(C,N), where x = 1.5 for methyl H and x = 1.2 for all other H atoms.

### Crystal data

**Table:**

C_11_H_15_NO_2_	*D*_x_ = 1.149 Mg m^−^^3^
*M**_r_* = 193.24	Melting point: 403K K
Orthorhombic, *P**b**c**a*	Mo *K*α radiation, λ = 0.71073 Å
Hall symbol: -P 2ac 2ab	Cell parameters from 25 reflections
*a* = 9.3010 (19) Å	θ = 9–12°
*b* = 7.6490 (15) Å	µ = 0.08 mm^−^^1^
*c* = 31.394 (6) Å	*T* = 294 K
*V* = 2233.5 (8) Å^3^	Block, colorless
*Z* = 8	0.30 × 0.10 × 0.10 mm
*F*(000) = 832	

### Data collection

**Table:**

Enraf–Nonius CAD-4 diffractometer	1099 reflections with *I* > 2σ(*I*)
Radiation source: fine-focus sealed tube	*R*_int_ = 0.0000
graphite	θ_max_ = 25.3°, θ_min_ = 1.3°
ω/2θ scans	*h* = 0→11
Absorption correction: ψ scan (North *et al.*, 1968)	*k* = 0→9
*T*_min_ = 0.977, *T*_max_ = 0.992	*l* = 0→37
2026 measured reflections	3 standard reflections every 120 min
2026 independent reflections	intensity decay: 1%

### Refinement

**Table:**

Refinement on *F*^2^	Secondary atom site location: difference Fourier map
Least-squares matrix: full	Hydrogen site location: inferred from neighbouring sites
*R*[*F*^2^ > 2σ(*F*^2^)] = 0.068	H-atom parameters constrained
*wR*(*F*^2^) = 0.202	*w* = 1/[σ^2^(*F*_o_^2^) + (0.1*P*)^2^] where *P* = (*F*_o_^2^ + 2*F*_c_^2^)/3
*S* = 1.01	(Δ/σ)_max_ < 0.001
2026 reflections	Δρ_max_ = 0.26 e Å^−^^3^
127 parameters	Δρ_min_ = −0.23 e Å^−^^3^
Primary atom site location: structure-invariant direct methods	

### Special details

**Table:**

Geometry. All esds (except the esd in the dihedral angle between two l.s. planes) are estimated using the full covariance matrix. The cell esds are taken into account individually in the estimation of esds in distances, angles and torsion angles; correlations between esds in cell parameters are only used when they are defined by crystal symmetry. An approximate (isotropic) treatment of cell esds is used for estimating esds involving l.s. planes.
Refinement. Refinement of F^2^ against ALL reflections. The weighted R-factor wR and goodness of fit S are based on F^2^, conventional R-factors R are based on F, with F set to zero for negative F^2^. The threshold expression of F^2^ > 2sigma(F^2^) is used only for calculating R-factors(gt) etc. and is not relevant to the choice of reflections for refinement. R-factors based on F^2^ are statistically about twice as large as those based on F, and R- factors based on ALL data will be even larger.

### Fractional atomic coordinates and isotropic or equivalent isotropic displacement parameters (Å^2^)

**Table:**

	*x*	*y*	*z*	*U*_iso_*/*U*_eq_	
N	0.5437 (3)	0.2110 (3)	0.52353 (8)	0.0516 (7)	
H0A	0.4609	0.2564	0.5183	0.062*	
O1	0.5722 (3)	−0.0692 (3)	0.68713 (7)	0.0834 (8)	
O2	0.7614 (2)	0.1579 (3)	0.49473 (7)	0.0665 (7)	
C1	0.5187 (7)	−0.3739 (7)	0.67643 (15)	0.128 (2)	
H1A	0.5192	−0.3613	0.6460	0.192*	
H1B	0.4231	−0.3546	0.6870	0.192*	
H1C	0.5494	−0.4897	0.6839	0.192*	
C2	0.6254 (7)	−0.2587 (7)	0.74329 (13)	0.1163 (17)	
H2A	0.6915	−0.1733	0.7542	0.175*	
H2B	0.6577	−0.3737	0.7510	0.175*	
H2C	0.5318	−0.2385	0.7552	0.175*	
C3	0.6181 (5)	−0.2437 (6)	0.69566 (12)	0.0810 (12)	
H3A	0.7146	−0.2607	0.6838	0.097*	
C4	0.5728 (4)	−0.0080 (4)	0.64580 (11)	0.0602 (9)	
C5	0.6698 (3)	−0.0586 (4)	0.61512 (10)	0.0589 (9)	
H5A	0.7396	−0.1415	0.6215	0.071*	
C6	0.6637 (3)	0.0134 (4)	0.57479 (10)	0.0543 (8)	
H6A	0.7297	−0.0221	0.5543	0.065*	
C7	0.5608 (3)	0.1378 (4)	0.56435 (9)	0.0470 (8)	
C8	0.4652 (4)	0.1886 (5)	0.59609 (12)	0.0631 (10)	
H8A	0.3955	0.2722	0.5900	0.076*	
C9	0.4712 (4)	0.1183 (5)	0.63610 (11)	0.0680 (10)	
H9A	0.4069	0.1555	0.6569	0.082*	
C10	0.6390 (3)	0.2191 (4)	0.49183 (10)	0.0515 (8)	
C11	0.5892 (4)	0.3070 (5)	0.45170 (11)	0.0638 (10)	
H11A	0.6652	0.3047	0.4310	0.096*	
H11B	0.5638	0.4260	0.4578	0.096*	
H11C	0.5069	0.2464	0.4406	0.096*	

### Atomic displacement parameters (Å^2^)

**Table:**

	*U*^11^	*U*^22^	*U*^33^	*U*^12^	*U*^13^	*U*^23^
N	0.0428 (13)	0.0549 (16)	0.0572 (16)	0.0014 (12)	−0.0018 (11)	0.0035 (13)
O1	0.119 (2)	0.0703 (17)	0.0608 (16)	0.0233 (16)	0.0052 (14)	0.0019 (13)
O2	0.0471 (13)	0.0715 (16)	0.0808 (16)	0.0047 (12)	0.0103 (12)	0.0115 (12)
C1	0.198 (6)	0.092 (4)	0.094 (4)	−0.034 (4)	0.005 (4)	0.003 (3)
C2	0.168 (5)	0.107 (4)	0.074 (3)	0.024 (4)	0.001 (3)	0.022 (3)
C3	0.096 (3)	0.069 (2)	0.078 (3)	0.014 (2)	0.008 (2)	0.011 (2)
C4	0.076 (2)	0.0508 (19)	0.054 (2)	0.0052 (19)	−0.0027 (17)	−0.0050 (16)
C5	0.061 (2)	0.051 (2)	0.065 (2)	0.0133 (17)	−0.0040 (16)	0.0000 (17)
C6	0.0534 (18)	0.0518 (19)	0.058 (2)	0.0022 (16)	0.0051 (15)	−0.0014 (16)
C7	0.0428 (15)	0.0439 (17)	0.0543 (19)	−0.0027 (14)	−0.0004 (13)	−0.0027 (14)
C8	0.062 (2)	0.057 (2)	0.071 (2)	0.0136 (17)	0.0049 (17)	0.0004 (18)
C9	0.076 (2)	0.064 (2)	0.063 (2)	0.017 (2)	0.0114 (17)	−0.0049 (19)
C10	0.0486 (17)	0.0420 (18)	0.064 (2)	−0.0084 (15)	0.0008 (15)	−0.0044 (15)
C11	0.061 (2)	0.063 (2)	0.067 (2)	−0.0106 (18)	−0.0042 (16)	0.0080 (18)

### Geometric parameters (Å, °)

**Table:**

N—C10	1.334 (4)	C4—C5	1.375 (4)
N—C7	1.407 (4)	C4—C9	1.385 (4)
N—H0A	0.8600	C5—C6	1.382 (4)
O1—C4	1.379 (4)	C5—H5A	0.9300
O1—C3	1.427 (5)	C6—C7	1.389 (4)
O2—C10	1.234 (4)	C6—H6A	0.9300
C1—C3	1.487 (6)	C7—C8	1.391 (4)
C1—H1A	0.9600	C8—C9	1.367 (5)
C1—H1B	0.9600	C8—H8A	0.9300
C1—H1C	0.9600	C9—H9A	0.9300
C2—C3	1.501 (5)	C10—C11	1.501 (4)
C2—H2A	0.9600	C11—H11A	0.9600
C2—H2B	0.9600	C11—H11B	0.9600
C2—H2C	0.9600	C11—H11C	0.9600
C3—H3A	0.9800		
			
C10—N—C7	128.5 (3)	C4—C5—C6	120.2 (3)
C10—N—H0A	115.7	C4—C5—H5A	119.9
C7—N—H0A	115.7	C6—C5—H5A	119.9
C4—O1—C3	119.5 (3)	C5—C6—C7	121.2 (3)
C3—C1—H1A	109.5	C5—C6—H6A	119.4
C3—C1—H1B	109.5	C7—C6—H6A	119.4
H1A—C1—H1B	109.5	C6—C7—C8	117.6 (3)
C3—C1—H1C	109.5	C6—C7—N	124.4 (3)
H1A—C1—H1C	109.5	C8—C7—N	118.0 (3)
H1B—C1—H1C	109.5	C9—C8—C7	121.5 (3)
C3—C2—H2A	109.5	C9—C8—H8A	119.3
C3—C2—H2B	109.5	C7—C8—H8A	119.3
H2A—C2—H2B	109.5	C8—C9—C4	120.3 (3)
C3—C2—H2C	109.5	C8—C9—H9A	119.9
H2A—C2—H2C	109.5	C4—C9—H9A	119.9
H2B—C2—H2C	109.5	O2—C10—N	122.7 (3)
O1—C3—C1	111.3 (4)	O2—C10—C11	121.1 (3)
O1—C3—C2	105.8 (3)	N—C10—C11	116.2 (3)
C1—C3—C2	112.4 (4)	C10—C11—H11A	109.5
O1—C3—H3A	109.1	C10—C11—H11B	109.5
C1—C3—H3A	109.1	H11A—C11—H11B	109.5
C2—C3—H3A	109.1	C10—C11—H11C	109.5
C5—C4—O1	124.5 (3)	H11A—C11—H11C	109.5
C5—C4—C9	119.3 (3)	H11B—C11—H11C	109.5
O1—C4—C9	116.1 (3)		
			
C4—O1—C3—C1	−65.9 (5)	C10—N—C7—C6	−21.2 (5)
C4—O1—C3—C2	171.7 (4)	C10—N—C7—C8	161.2 (3)
C3—O1—C4—C5	−32.2 (5)	C6—C7—C8—C9	−0.4 (5)
C3—O1—C4—C9	150.6 (4)	N—C7—C8—C9	177.4 (3)
O1—C4—C5—C6	−178.5 (3)	C7—C8—C9—C4	−0.8 (5)
C9—C4—C5—C6	−1.3 (5)	C5—C4—C9—C8	1.7 (5)
C4—C5—C6—C7	0.2 (5)	O1—C4—C9—C8	179.0 (3)
C5—C6—C7—C8	0.7 (5)	C7—N—C10—O2	0.6 (5)
C5—C6—C7—N	−176.9 (3)	C7—N—C10—C11	−179.7 (3)

### Hydrogen-bond geometry (Å, °)

**Table:**

*D*—H···*A*	*D*—H	H···*A*	*D*···*A*	*D*—H···*A*
N—H0A···O2^i^	0.86	2.01	2.869 (3)	175
C6—H6A···O2	0.93	2.34	2.892 (4)	118

Symmetry codes: (i) *x*−1/2, −*y*+1/2, −*z*+1.
